# The influence of periampullary diverticula on ERCP for treatment of common bile duct stones

**DOI:** 10.1038/s41598-020-68471-8

**Published:** 2020-07-10

**Authors:** Yang Hu, Da-Qing Kou, Shi-Bin Guo

**Affiliations:** 1 Department of Gastroenterological Endoscopy, The First Affiliated Hospital of Dalian Medical University, 222 Zhongshan road, Xigang district, Dalian, 116011 Liaoning China; 2 Department of Clinical Laboratory, The First Affiliated Hospital of Dalian Medical University, Dalian, 116011 Liaoning China

**Keywords:** Choledocholithiasis, Oesophagogastroscopy

## Abstract

In order to evaluate the effectiveness of various methods we applied to decrease the influence of periampullary diverticula (PAD) on the success rate and complications of endoscopic retrograde cholangiopancreatography (ERCP) in the treatment of common bile duct (CBD) stones, we enrolled patients with CBD stones who had been treated by ERCP in our hospital between January 2015 and December 2018. According to the presence of PAD, the patients were divided into a PAD group and a non-PAD group. The rate of complete stone removal in the first session, the rate of overall stone removal, the frequency of application of mechanical lithotripsy, and procedure-related complications, including bleeding, hyperamylasemia, pancreatitis, perforation, and infection of biliary tract were recorded. A total of 183 cases, including 72 cases in the PAD group and 111 cases in the non-PAD group were enrolled. There was no statistical difference between the two groups regarding gender (P = 0.354). However, regarding age, there was a statistical difference (P = 0.002), and the incidence of PAD increased with age. There were 5 (6.9%) cases in the PAD group and 14 (12.6%) cases in the non-PAD group where mechanical lithotripsy was applied. There were 59 (81.9%) cases in the PAD group and 102 (91.9%) cases in the non-PAD group where there was complete removal of CBD stones in the first session, and there were 68 (94.4%) cases in the PAD group and 107 (96.4%) cases in the non-PAD group where there was complete removal of all stones. In the PAD group, there were 0 cases (0%) with gastrointestinal bleeding, 0 cases (0%) with gastrointestinal perforation, 13 cases (18.1%) with post-ERCP hyperamylasemia, 3 cases (4.2%) with post-ERCP pancreatitis, and 4 cases (5.6%) with biliary tract infection. In the non-PAD group, 1 case (0.9%) had gastrointestinal bleeding, 0 cases had gastrointestinal perforation, 18 cases (16.2%) had post-ERCP hyperamylasemia, 5 cases (4.5%) had post-ERCP pancreatitis, and 11 cases (9.9%) had biliary tract infection. This retrospective study showed that there was a statistical difference between the two groups regarding complete removal of CBD stones in the first session and application of mechanical lithotripsy (both P < 0.05), but no statistical difference according to the rates of overall stone removal and the complications (P > 0.05), which means that we can reduce the influence of PAD on ERCP for treatment of common bile duct stones.

## Introduction

The periampullary diverticulum (PAD) is an outpouching herniation in the duodenal wall near the major duodenal papilla^1^. Most PAD is asymptomatic, found incidentally by CT or ERCP^2,3^. However, PAD may cause of the sphincter of Oddi dysfunction, allowing reflux of pancreatic and intestinal juice into the bile duct. Moreover, PAD could mechanically compress the distal portion of the common bile duct, as well as cause papillary spasm, leading to bile stasis and formation of common bile duct (CBD) stones^4–7^. Previous studies showed that there was a close correlation between PAD and the formation of CBD stones^8–10^.

Therapeutic ERCP, widely accepted as the standard therapy for removing CBD stones, could be difficult and have higher rates of complications in patients when accompanied by PAD, because the location and orientation of the major duodenal papilla may be changed. This retrospective study aimed to evaluate the effectiveness of various methods we applied to reduce the influence of PAD on the success rate and complications of ERCP in the treatment of CBD stones.

## Materials and methods

### Patients

**Figure 1:**
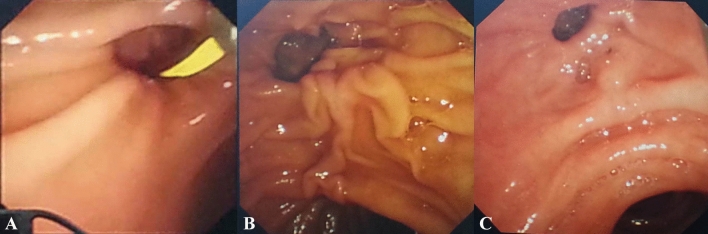
The locations of the major papilla with diverticula. (**A**) Papilla was located inside the diverticula; (**B**) papilla was located at the margin of the diverticula; (**C**) papilla was located outside the diverticula.

Patients with CBD stones, treated by ERCP in our hospital from January 2015 to December 2018, were retrospectively reviewed. Patients’ exclusion criteria were any of the following: history of EST, surgical history involving the gastrointestinal tract, co-existing bile leakage, choledochoduodenal fistula, severe bleeding tendency, concomitant pancreatic or biliary malignant disorders. According to the presence of PAD, the patients were divided into a PAD group and a non-PAD group. PAD was sub-classified under two types based on the locations of the major papilla: type A, where the papilla was located outside the diverticula or at the margin of the diverticula, and type B, where the papilla was located inside the diverticula^11^ (Fig. 1). The study was executed according to the Helsinki Declaration and followed the local legislation and was approved by the Ethics Committee of First Hospital affiliated to Dalian Medical University. All the patients or their relatives presented written informed consent before the procedure.

### Methods

Before the procedure, blood samples were collected for coagulation status; an abdominal CT or MRI was also performed. The liver-function tests (alanine aminotransferase, aspartate aminotransferase, bilirubin, alkaline phosphatase, and γ-glutamyl transpeptidase) and routine blood tests were performed before the procedure and the morning after, as well as serum amylase testing (at 3 h, 6 h and 24 h, respectively)^12^. All results were documented.

**Figure 2:**
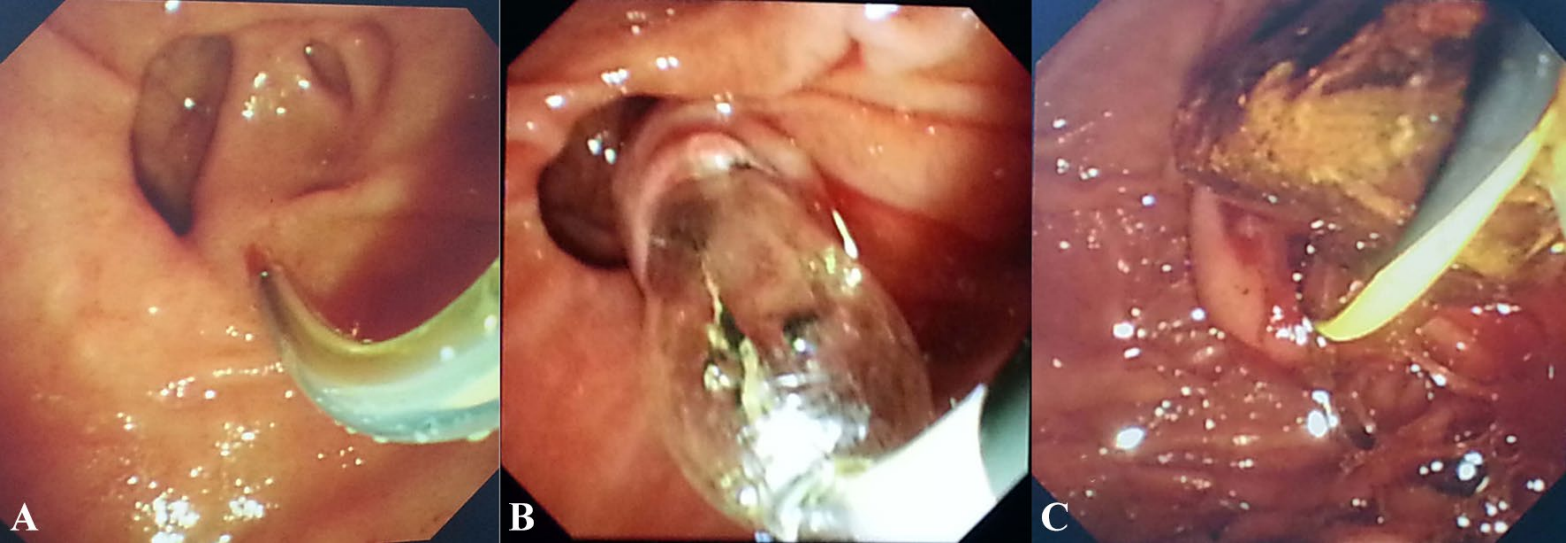
Endoscopic view of removal of CBD stones. (**A**) Selective cannulation of the CBD; (B) endoscopic papillary balloon dilation; (**C**) a large stone extracted using a retrieval balloon catheter through the dilated papilla.

**Figure 3:**
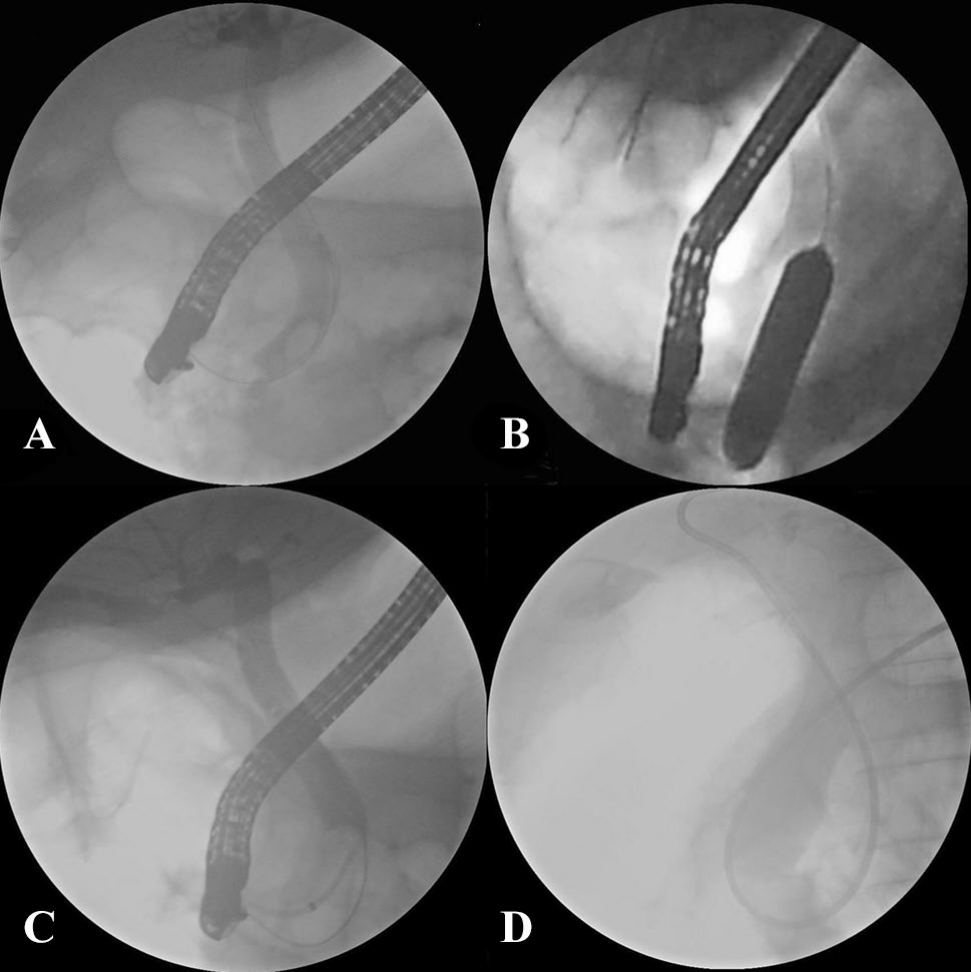
Fluoroscopic view of removal of CBD stones. (**A**) Cholangiogram demonstrating CBD stone; (**B**) a balloon inflated across the papilla over the guidewire; (**C**) the cholangiogram following complete stone removal showed no residual filling defect in CBD; (**D**) the placement of a nasobiliary drainage tube.

Some procedures were accomplished under ECG monitoring. Tetracaine was used for local anesthesia of the pharynx. The patients were sedated and given pain relievers via intramuscular injection of diazepam (5 mg) and meperidine (50 mg). Administrations of 20 mg of butyl scopolamine bromide were given intramuscularly prior to the procedure to inhibit duodenal peristalsis. All these procedures were performed by experienced endoscopists at a single center with side-viewing endoscopes (JF-260; Olympus Optical Corporation, Tokyo, Japan) (Figs. 2, 3).

Selective cannulation of the CBD was performed by a sphincterotome with a guidewire. The double-guidewire technique or the transpancreatic precut was applied in some difficult cases, such as inflammation or special morphology of papilla, and periampullary diverticula. For cases with CBD stones embedded within the papilla, needle-knife papillotomy was performed. For type B diverticula, we first exposed the papilla by eversion using biopsy forceps, or fixation by metal clip, or submucosal injection of saline, and the double-guidewire technique was often applied.

After cannulation of the CBD and cholangiography, endoscopic sphincterotomy (EST) alone or small EST combined with endoscopic papillary balloon dilation (EPBD) was performed. For cases with either abnormal coagulation (because of taking antiplatelet and anticoagulation medicine) or with type B diverticula, EPBD alone was performed.

Mechanical lithotripsy was applied when CBD stones were too large. However, if a patient was in poor condition or had large CBD stones with type B diverticula, endoscopic retrograde biliary drainage (ERBD) alone was performed. Three months later, when stones would have become smaller and softer, a subsequent ERCP was performed for removal of CBD stones.

The guidewire was always kept in bile duct during the whole procedure, and in most cases endoscopic nasobiliary drainage (ENBD) was placed after removal of CBD stones, especially in cases with mechanical lithotripsy. For cases with multiple pancreatic-duct cannulations, endoscopic retrograde pancreatic drainage (ERPD) was performed.

The collected data included the cases of the first-session complete stone removal and cases of overall stone removal, the frequency of application of mechanical lithotripsy, and associated complications of the procedure (including bleeding, hyperamylasemia, pancreatitis, perforation, and infection of biliary tract). Post-ERCP pancreatitis (PEP) is defined as abdominal pain lasting for over 24 h with a level of serum amylase exceeding three times the normal upper limit (NUL)^13^. Hyperamylasemia is defined as a serum amylase level more than three times the NUL without pain in the abdomen^13^. Post-ERCP bleeding is categorized as major or minor according to the quantity of hemorrhage. If it is severe hemorrhage requiring transfusion or interventions, then it’s major bleeding, while minor bleeding is self-limited or mild hemorrhage that can be controlled using an endoscope, so transfusion is not needed^14^. Cholangitis is defined as a fever accompanied with post-procedure right-upper-quadrant pain and leukocytosis^14^. We retrospectively evaluated both clinical and endoscopic data.

### Statistical analysis

We performed the data analyses using the Statistical SPSS 19.0 software (Chicago, IL, USA), and compared the categorical parameters using the chi-square test or Fisher’s exact test, and compared the continuous variables using the Student’s t-test. All the measurements in our study were shown as mean ± standard deviation (SD). *P* < 0.05 was considered statistical significance.

## Results

1. The relationship between PAD and gender, age.

The characteristics of the 183 patients (99 male, 84 female; age range from 23 to 94 years) relative to demography are shown in Table 1. There was no difference in statistics between the PAD group and the non-PAD group relative to gender. Within the PAD group, the age range was from 28 to 94 years, with an average 71, while within the non-PAD group, age ranged from 23 to 92 years, with an average 61. There was a difference in statistics between the PAD group and non-PAD group relative to age, and the incidence of PAD increased with age (Table 1).

2. The relationship between PAD and CBD stones.

**Table 1:** Demographic characteristics of patients in PAD group and non-PAD group.

	PAD group (n = 72)	Non-PAD group (n = 111)	P value
**Gender**			
Male	42 (58.3%)	57 (51.4%)	
Female	30 (41.7%)	54 (48.6%)	0.354
**Age**			
< 40	1 (1.4%)	15 (13.5%)	
40 ~ 49	5 (6.9%)	11 (9.9%)	
50 ~ 59	7 (9.7%)	23 (20.7%)	
60 ~ 69	18 (25.0%)	25 (22.5%)	
≥ 70	41 (56.9%)	37 (33.3%)	0.002
**CBD**			
Mean diameter of stones (mm)	12.5 ± 3.6 (5–22)	12.8 ± 3.8 (4–36)	0.590
Mean number of stones	2.2 ± 1.4	2.3 ± 1.5	0.647
Mean diameter of CBD (mm)	13.4 ± 3.5 (7–23)	13.6 ± 3.8 (8–37)	0.715
**Number**			
< 3	58 (80.6%)	90 (81.1%)	
≥ 3	14 (19.4%)	21 (18.9%)	0.930
**Size**			
< 1 cm	42 (58.3%)	72 (64.9%)	
1 ~ 2 cm	25 (34.7%)	34 (30.6%)	
≥ 2 cm	5 (7.0%)	5 (4.5%)	0.606
**Context**			
Jaundice	29 (40.2%)	44 (39.6%)	0.931
Abnormal liver function	21 (29.2%)	32 (28.8%)	0.961
Cholangitis	12 (16.7%)	21 (18.9%)	0.699
Biliary pancreatitis	5 (6.9%)	8 (7.2%)	0.946
Aspirin/anticoagulants	6 (8.3%)	8 (7.2%)	0.779
**Comorbidity**			
CAD	10 (13.9%)	17 (15.3%)	0.790
COPD	3 (4.2%)	5 (4.5%)	0.913

*CAD* coronary artery disease, *COPD* chronic obstructive pulmonary disease.

There were 1–8 CBD stones found, and the largest diameter of a stone was 3.6 cm. The mean stone size in the 183 patients was 12.7 ± 3.4 mm (range 5–36 mm), and the mean bile-duct diameter was 13.6 ± 3.5 mm (range 8–36 mm). There was no statistical difference between the two groups relative to size (12.5 ± 3.6 mm vs*.* 12.8 ± 3.8 mm, *P* > 0.05), number of stones (2.2 ± 1.4 vs*.* 2.3 ± 1.5, *P* > 0.05), or diameters of CBD (13.4 ± 3.5 mm vs*.* 13.6 ± 3.8 mm, *P* > 0.05).

There were 73 cases with jaundice; among them, 29 cases were in the PAD group. There were 53 cases with abnormal liver function, among them, 21 cases had diverticula. There were 33 cases with cholangitis, among them, 12 cases were in the PAD group. There were 13 cases with biliary pancreatitis, among them, 5 cases had diverticula. There were 14 cases with abnormal coagulation status, among them, 6 cases were in in the PAD group.

Some cases had comorbidity: 27 cases had coronary artery disease, among them, 10 cases had diverticula; 8 cases had chronic obstructive pulmonary disease, among them, 3 cases were in the PAD group.

3. The influence of PAD on ERCP

The procedures relative to the two groups are detailed in Table 2.

**Table 2:** Comparison of procedure, success and complications between the two groups.

	PAD group (n = 72)	Non-PAD group (n = 111)	P value
**Procedural**			
Double-guidewire technique	11 (15.3%)	16 (14.4%)	0.872
Transpancreatic precut	3 (4.2%)	5 (4.5%)	0.913
Needle knife application	0 (0%)	2 (1.8%)	0.252
Failure cannulation of CBD	1 (1.4%)	1 (0.9%)	0.756
EST alone	23 (31.9%)	47 (42.3%)	0.157
EPBD alone	8 (11.1%)	10 (9.0%)	0.641
Small EST + EPBD	33 (45.8%)	49 (43.2%)	0.822
ERPD	7 (9.7%)	15 (13.5%)	0.441
ERBD	8 (11.1%)	5 (4.5%)	0.089
ENBD	61 (84.7%)	103 (92.8%)	0.080
Frequency of mechanical lithotripsy	5 (6.9%)	14 (12.6%)	0.035
**Result**			
Complete stone removal in 1st session	59 (81.9%)	102 (91.9%)	0.043
Overall stone removal	68 (94.4%)	107 (96.4%)	0.528
**Complications**			
Bleeding minor bleeding	0 (0%)	1 (0.9%)	0.419
Major bleeding	0 (0%)	0 (0%)	NA
Perforation	0 (0%)	0 (0%)	NA
Hyperamylasemia	13 (18.1%)	18 (16.2%)	0.746
Pancreatitis	3 (4.2%)	5 (4.5%)	0.913
Infection of biliary tract	4 (5.6%)	11 (9.9%)	0.294

In the PAD group, there were 59 cases (81.9%) in which the CBD stones were removed completely in the first session. Among the remaining 13 cases (18.1%), one was a case of failure in cannulation of the CBD because of difficulty in detecting the papilla, nine cases had too many and/or too large of CBD stones to be completely removed in the first session, and three cases had stenosis of the lower part of CBD. In the non-PAD group, there were 102 cases (91.9%) in which the CBD stones were removed completely in the first session. Among the remaining 9 cases (8.1%), there was 1 case of failure of cannulation of CBD, eight cases of too many and/or too large of CBD stones to be completely removed in the first session. There was a difference in statistics between the two groups concerning complete removal of CBD stones in the first session (81.9% vs. 91.9%, P < 0.05) and the frequency of mechanical lithotripsy [5/72 (6.9%) vs. 14/111 (12.6%), P < 0.05], but no statistical difference according to the rate of overall stone removal [68/72 (94.4%) vs. 107/111 (96.4%), P > 0.05].

In the PAD group, there was no bleeding associated with ERCP, while in the non-PAD group, bleeding occurred in 1 case. There was no statistical difference between the two groups concerning hemorrhage.

No perforation cases existed in either group.

In the PAD group, hyperamylasemia occurred in 13 cases (18.1%), while in the non-PAD group, hyperamylasemia occurred in 18 cases (16.2%). There was no difference in statistics between the two groups concerning hyperamylasemia.

In the PAD group, PEP occurred in 3 cases (4.2%), while in the non-PAD group, PEP occurred in 5 cases (4.5%). There was no statistical difference between the two groups concerning PEP.

In the PAD group, infection of the biliary duct occurred in 4 cases (5.6%), while in the non-PAD group, infection of the biliary duct occurred in 11 cases (9.9%). There was no difference in statistics between the two groups concerning biliary duct infection.

4. The basic information of patients in type A and type B PAD.

Sub-analysis by PAD type showed that there was a statistical difference concerning the number of CBD stones between the type A PAD group and type B PAD group, but no statistical difference relative to the size of CBD stones (Table 3).

**Table 3:** Demographic characteristics of patients in type A and type B PAD.

	Type A PAD (n = 58)	Type B PAD (n = 14)	P value
**Gender**			
Male	34 (58.6%)	8 (57.1%)	
Female	24 (41.4%)	6 (42.9%)	0.920
**Age**			
<40	1 (1.7%)	0 (0%)	
40 ~ 49	5 (8.6%)	0 (0%)	
50 ~ 59	4 (6.9%)	3 (21.4%)	
60 ~ 69	10 (17.2%)	8 (57.1%)	
≥ 70	38 (65.5%)	3 (21.4%)	0.005
**CBD**			
Mean diameter of stones (mm)	12.4 ± 3.4 (5–22)	9.5 ± 3.2 (7–15)	0.007
Mean number of stones	2.2 ± 1.5	1.6 ± 1.3	0.147
Mean diameter of CBD (mm)	13.5 ± 3.4 (7–23)	10.2 ± 3.5 (8–16)	0.005
**Number**			
< 3	51 (87.9%)	7 (50.0%)	
≥ 3	7 (12.1%)	7 (50.0%)	0.001
**Size**			
< 1 cm	34 (58.6%)	8 (57.1%)	
1 ~ 2 cm	19 (32.8%)	6 (42.9%)	
≥ 2 cm	5 (8.6%)	0 (0%)	0.462
**Context**			
Jaundice	24 (41.3%)	5 (35.7%)	0.698
Abnormal liver function	17 (29.3%)	4 (28.6%)	0.956
Cholangitis	10 (17.2%)	2 (14.3%)	0.790
Biliary pancreatitis	4 (6.9%)	1 (7.1%)	0.974
Aspirin/anticoagulants	4 (6.9%)	2 (14.3%)	0.369
**Comorbidity**			
CAD	8 (13.8%)	2 (14.3%)	0.962
COPD	3 (5.2%)	0 (0%)	0.385

The characteristics of patients in the two subtypes, the procedures and the results including complications were listed in the Tables 3 and 4.

**Table 4:** The procedure, rates of success and complications in type A and type B PAD.

	Type A PAD (n = 58)	Type B PAD (n = 14)	P value
**Procedural**			
Double-guidewire technique	8 (13.8%)	3 (21.4%)	NA
Transpancreatic precut	3 (5.2%)	0 (0%)	NA
Needle knife application	0 (0%)	0 (0%)	NA
Failure cannulation of CBD	0 (0%)	1 (7.1%)	NA
EST alone	23 (39.7%)	0 (0%)	NA
EPBD alone	4 (6.9%)	4 (28.6%)	NA
Small EST + EPBD	27 (46.6%)	6 (42.9%)	NA
ERPD	5 (8.6%)	2 (14.3%)	NA
ERBD	5 (8.6%)	3 (21.4%)	NA
ENBD	50 (86.2%)	11 (78.6%)	NA
Frequency of mechanical lithotripsy	5 (8.6%)	0 (0%)	NA
**Result**			
Complete stone removal in 1st session	49 (84.5%)	10 (71.4%)	NA
Overall stone removal	56 (96.6%)	12 (85.7%)	NA
**Complications**			
Bleeding minor bleeding	0 (0%)	0 (0%)	NA
Major bleeding	0 (0%)	0 (0%)	NA
Perforation	0 (0%)	0 (0%)	NA
Hyperamylasemia	10 (17.2%)	3 (21.4%)	NA
Pancreatitis	2 (3.4%)	1 (7.1%)	NA
Infection of biliary tract	4 (6.9%)	0 (0%)	NA

## Discussion

There are some limitations in this study, such as it is a retrospective study, and the sample size is small.

The duodenal diverticulum is a mucosal or submucosal outpouching with partly weak muscle along the intestinal wall^15^. It is usually located in the second part of the duodenum. The origin and development of PAD include: congenital factors, and acquired factors, including old age, progression of duodenal motility disorders, progressive weakening of intestinal smooth muscles, increased intraduodenal pressure and the sphincter of Oddi dysfunction^16^. The incidence of PAD is infrequent before the age of 30 years^17^. It’s usually found in the elderly, slightly more frequently in females than in males^18^. In our study, the rate of PAD increases with age, but is not related to gender. The type of PAD did not vary significantly according to age or gender.

Many studies show that PAD is associated with increased frequency of pancreatobiliary diseases^9^. The study of Rajnakova et al. shows that patients with PAD were 1.8 times more likely to have CBD stones, compared with patients without PAD^19^. Kennedy and Thompson also reported that patients having CBD stones were 2.6 times more likely to have PAD than those without^20^. PAD may influence the success rate of therapeutic or diagnostic ERCP procedures^21^. Some studies showed that the existence of PAD had an association with cannulation difficulty during ERCP^21^, and is related to higher risks of complication^19^. Failures in cannulation may be partially attributable to difficulties in detecting the papilla, especially in cases where the papilla is found deep within, often at the very bottom of, the diverticulum^16^. But in some other studies, the presence of PAD had no influence on cannulation of CBD^22^. The different results may be explained by various techniques for cannulation and different types of PAD. In our study, many methods were used to expose the papilla, such as eversion of the diverticulum by means of biopsy forceps or fixation with a metal clip, or submucosal injection of saline, indwelling of a guide wire in the pancreatic duct or by placement of a pancreatic duct stent.

Because of the abnormal anatomical structure of the diverticulum, the risk of bleeding and perforation caused by large endoscopic sphincterotomy (EST) or mechanical lithotripsy is very high.

Since mechanical lithotripsy takes a long time for large and hard CBD stones, and some patients with PAD in our studies were old and in poor condition, they could not tolerate the long procedure time. In this situation, ERBD alone was performed for drainage and to relieve symptoms. Three months later, the stones, having become smaller and softer, were easily removed. Although these treatments decreased the success rate of ERCP lithotomy in the first session, they also decreased the ERCP-associated complications. In our study, the existence of PAD had no influence on the deep cannulation of CBD, but decreased the rate of complete CBD stone removal in the first session.

PEP is one of the most feared complications of ERCP. It occurs in 5–19.8% of patients after endoscopic papillary balloon dilation (EPBD)^23^. The balloon dilation of the sphincter of Oddi may cause compression, spasm and edema of the distal pancreatic duct, leading to the restriction of pancreatic juice flow and the occurrence of pancreatitis^24^. It is reported that, compared to EPBD alone, small EST combined with EPBD can reduce the risk of PEP by guiding the orientation of the dilation towards the CBD, which prevents pressure overburden on the main pancreatic duct^25^. Moreover, large openings of the bile duct in association with large balloon dilations could eliminate unintended pancreatic-duct cannulizations in ensuing stone extractions. The guide wire was kept in the CBD for direction. This is very important, especially for type B PAD, since predicting the direction of the bile duct is very difficult. Another key point to decrease the incidence of PEP is selective cannulization of the CBD when performing the ERCP^26^. In our study, we used a sphincterotome with a guide wire instead of a catheter, to avoid injecting contrast medium into the pancreatic duct. Our study showed that there was no significant difference between the two groups concerning PEP and hyperamylasemia, and that all cases of pancreatitis were mild, recovering after conservative treatment in less than 72 h.

Regarding the risk of hemorrhage, EPBD has its own advantages: less trauma to the ampullary sphincter and less bleeding^27^. Since compression by a balloon is an effective haemostasis, small EST combined with EPBD could reduce procedure-related hemorrhage. For cases when antiplatelet and/or anticoagulation medicine was being taken, EPBD alone was performed before stone removal, or only ERBD initially performed, with stone removal being performed one week after. In our study, there was one case of bleeding in the non-PAD group (minor bleeding, stopped via administration of hemostatic agents). There was no significant difference between the two groups concerning procedure-related hemorrhage. Although some studies reported that there was a higher rate of bleeding in EPBD combined with EST groups, we attributed those results to the moderate degree of EST.

Perforation is another fatal complication of therapeutic ERCP. Since PAD consists of thin mucosa lacking smooth muscle^28^, it may increase the potential risks of perforation during therapeutic ERCP procedures. In this case, the length of EST is relatively short, and outcomes of therapeutic ERCP were influenced^19^. However, during balloon dilation after small EST, the endoscopists could observe the dilation status of the ampulla by endoscopy and fluoroscopy, and the risk of perforation thereby could be reduced. To minimize the risk of perforation, the pressure of the balloon should be increased gradually, and the size of the dilated balloon should not exceed the size of the CBD. If the CBD stones are too large in patients with type B PAD, ERBD was performed for drainage and to relieve symptoms, and thereafter therapeutic ERCP was performed 3 months later. Consequently, there were no cases of perforation in either group in our study. Moreover, the combination of EPBD with small EST provided a spacious opening of the bile duct, reducing the need for mechanical lithotripsy^29^.

Cholangitis is an infective complication of ERCP^30^, jaundice and inadequate drainage of the biliary duct system were regarded as independent risk factors^31^. In addition to adequate biliary duct drainage, antibiotic prophylaxis can also reduce bacteremia and seems to prevent cholangitis in patients undergoing ERCP^32^, and is recommended by American Gastrointestinal Endoscopy guidelines and British Society of Gastroenterology guidelines for routine antibiotic prophylaxis prior to ERCP^33,34^. In our study, there were 4 cases (5.6%) in the PAD group and 11 cases (9.9%) in the non-PAD group with infection of biliary duct system, higher than previous reports^35^. The reasons can be summarized as follows: most of the cases had acute or chronic cholangitis, cholecystitis, obstructive jaundice, fever, elevated leukocyte counts and elevated neutrophil–lymphocyte ratio before the performance of ERCP. Incomplete drainage after mechanical lithotripsy is another reason. There was no significant difference between the two groups concerning infection of the biliary duct system in our study, and all these cases were mild, recovering less than 72 h after conservative treatments such as lavage and drainage through an ENBD tube and intravenous administration of antibiotics.

In summary, when performing ERCP with PAD, the double-guidewire technique is recommend. If the papilla was located inside the diverticulum, it should be fully exposed by eversion diverticulum. Small EST combined with EPBD was often performed before stone removal. For large CBD stones with type B diverticula, mechanical lithotripsy should be avoided. ERBD was done separately and a subsequent ERCP was performed for removal of CBD stones three months later. Employing these methods, we can achieve satisfactory results while reducing complications.

However, this was a retrospective study, which is one of its principal limitations. In addition, the small sample size may cause statistical bias, especially as the number of cases with type B diverticula is so small. The influence of the two subtypes of diverticula on ERCP should to be evaluated by future large randomized multicenter prospective case-controlled studies with a longer follow-up period.
